# In-situ diet–microbiota associations across taxonomic scales in desert-dwelling amphibians and reptiles

**DOI:** 10.1093/ismeco/ycaf213

**Published:** 2025-11-18

**Authors:** Wei Zhu, Ruoyao Ni, Bo Cai, Shun Ma, Jianping Jiang, Bin Wang

**Affiliations:** Chengdu Institute of Biology, Chinese Academy of Sciences, Chengdu 610213, Sichuan, China; College of Life Sciences, University of Chinese Academy of Sciences, Beijing 101408, China; Chengdu Institute of Biology, Chinese Academy of Sciences, Chengdu 610213, Sichuan, China; College of Life Sciences, University of Chinese Academy of Sciences, Beijing 101408, China; Chengdu Institute of Biology, Chinese Academy of Sciences, Chengdu 610213, Sichuan, China; Chengdu Institute of Biology, Chinese Academy of Sciences, Chengdu 610213, Sichuan, China; College of Life Sciences, University of Chinese Academy of Sciences, Beijing 101408, China; Chengdu Institute of Biology, Chinese Academy of Sciences, Chengdu 610213, Sichuan, China; College of Life Sciences, University of Chinese Academy of Sciences, Beijing 101408, China; Chengdu Institute of Biology, Chinese Academy of Sciences, Chengdu 610213, Sichuan, China; College of Life Sciences, University of Chinese Academy of Sciences, Beijing 101408, China

**Keywords:** arthropod, diet differentiation, microbiome, phylosymbiosis, resilience

## Abstract

Understanding how host and environmental factors shape gut microbiota is central to microbial ecology and evolution. However, the extent to which gut microbes covary with diet and how such variation reflects host phylogeny, remains unclear under natural conditions. Here, we used DNA metabarcoding of gut contents to analyze the dietary arthropod composition and gut microbiota of four amphibian and three reptile species from the Tarim Desert, Xinjiang, China. These species showed pronounced differences in both diet and microbial profiles. Dominant dietary arthropod families exhibited generally low overlap among species, and dietary variation did not align with host phylogeny. Interestingly, *Bufotes pewzowi* (amphibian) and *Teratoscincus przewalskii* (reptile)—the most common species in their respective groups—both primarily consumed ants (Formicidae). Conversely, gut microbial composition more closely reflected host phylogeny than diet, with a clear separation between amphibians and reptiles, particularly in the relative abundances of Bacteroidetes and the genera *Bacteroides* and *Blautia*. These findings suggest that the previously reported phylosymbiosis in these species is not primarily driven by dietary overlap. Significant diet–microbiota correlations were observed across all species and within each taxonomic class but were largely absent within species. This highlights taxonomic-level differences in the diet–microbiota relationship, indicating that diet-microbiota covariation is more pronounced over evolutionary timescales than in response to real-time dietary variation. Taken together, our results show that gut microbiota and diet exhibit distinct phylogenetic patterns, with microbiota showing both associations with diet and resilience to short-term dietary changes, underscoring the importance of considering timescales in diet–microbiota studies.

## Introduction

Dietary information is fundamental to ecological research, as it provides crucial insights into animals’ life history strategies, population dynamics, and their responses to environmental disturbances such as habitat degradation [1, 2]. Analyses of dietary composition are indispensable for understanding species’ evolution, local adaptation, distribution patterns, and long-term viability [3, 4]. From a conservation perspective, diet data offer essential context for management and restoration efforts [5], as the availability and composition of dietary resources can modulate the relationships between environmental factors and animal physiology, behavior, and fitness [6, 7].

As research increasingly underscores the critical role of gut microbiota in animal behavior, physiology, metabolism, and immunity [8–11], particularly in facilitating environmental adaptation and response [12, 13], unraveling the determinants of gut microbial composition has emerged as a focal point in ecology and conservation biology [14]. Diet fundamentally shapes the composition and function of gut microbiota across animal species [15, 16]. Extensive research has demonstrated that dietary differences strongly influence gut microbial diversity, stability, and metabolic potential [17–19]. For example, herbivorous animals harbor gut microbiota enriched with fiber-degrading bacteria (e.g. *Ruminococcus* and *Fibrobacter*), whereas carnivores exhibit a dominance of microbes associated with protein and fat metabolism (e.g. *Bacteroides* and *Fusobacterium*) [20]. In addition to evolutionary dietary specialization, dietary shifts within a single generation, such as seasonal fluctuations in food availability, can drive rapid changes in the composition and function of the gut microbiota [21, 22], highlighting the dynamic nature of diet-microbe interactions [23, 24]. Comparative studies across species suggest a co-evolutionary relationship between diet and gut microbiota [25], with specific microbial taxa consistently associated with particular dietary niches [20].

Despite the significant progress in our understanding on the associations between diet and gut microbes, many important questions remain unanswered. One major gap lies in understanding how diet influences gut microbiota at different taxonomic levels (e.g. species or higher taxonomic levels, populations, and individuals). Given that dietary differentiation plays a critical role in adaptive evolution and species formation, addressing this question could not only deepen our understanding of the mechanisms driving gut microbial diversity in vertebrates but also shed light on the evolution of microbial symbiosis. For instance, it could help determine whether diet mediates phylosymbiosis, a widely observed phenomenon in animals [25–28], where closely related host species tend to harbor more similar microbiomes than distantly related ones [27, 29]. Another critical gap concerns whether, and to what extent, individual gut microbial communities exhibit functional redundancy and resilience in response to dietary changes [30]. Most existing studies on these topics focus on laboratory-reared, homogenized individuals, examining their responses to dietary changes. In contrast, field studies investigating the responses of wild individuals to dietary fluctuations are limited, primarily due to the inherent challenges of continuously monitoring the diet of free-ranging animals and correlating it with the real-time dynamics of their gut microbiota.

Amphibians and reptiles serve as excellent models for addressing these questions, as a substantial proportion of them primarily consume arthropods [31, 32]. With the advancement of DNA barcoding technology, identifying insect composition from environmental samples has become highly efficient [33–35]. Studies have demonstrated that insect DNA barcoding based on gut contents or feces can accurately reflect the insect composition in the diet [36]. Therefore, we can simultaneously conduct amplicon sequencing of both gut microbiota and insect composition from gut contents, enabling real-time monitoring of dietary composition and gut microbiota dynamics in amphibians and reptiles. This integrated approach facilitates robust correlation analyses between dietary changes and gut microbial shifts. The Tarim Desert, situated in Xinjiang Uygur Autonomous Region in northwestern China, ranks among the largest and driest deserts globally, spanning around 350 000 km^2^. Its extreme aridity and harsh conditions are well-known. Despite these challenges, it hosts a unique range of fauna, including numerous reptiles and several amphibians, some with wide distribution ranges. Its vast expanse provides a natural laboratory for studying interspecies variations in diet and gut microbes, as well as testing their associations. We previously found phylosymbiosis in the amphibians and reptiles inhabiting the Tarim Desert [37], giving an opportunity to test the role of diet in shaping the host-microbe associations.

In this study, we employed amplicon sequencing to investigate the dietary arthropod and gut microbial composition of 222 gut content samples. These samples encompassed all four amphibian species present in the region (*Bufotes pewzowi*, *Bufotes taxkorensis*, *Pelophylax mongolius*, and *Pelophylax terentievi*) as well as three common reptile species (*Phrynocephalus axillaris*, *Tenuidactylus elongatus*, and *Teratoscincus przewalskii*). Differential analyses were conducted at both the class level (Amphibia vs. Reptilia) and the species level, while association analyses were performed between dietary arthropods and gut microbiota at the individual level. We hypothesize that (i) both dietary composition and gut microbiota differ significantly between animal classes and species, reflecting host phylogeny and thus explaining phylosymbiosis, (ii) significant associations exist between dietary composition and gut microbiota at the interspecies level but not within species, illustrating the role of diet in shaping gut microbiota and the resilience of gut microbes to dynamic dietary changes. We anticipate that these findings will enhance our understanding of macroevolutionary processes, species formation, population differentiation, and individual environmental adaptation.

## Materials and methods

### Sample collection and environmental information

**Figure 1:**
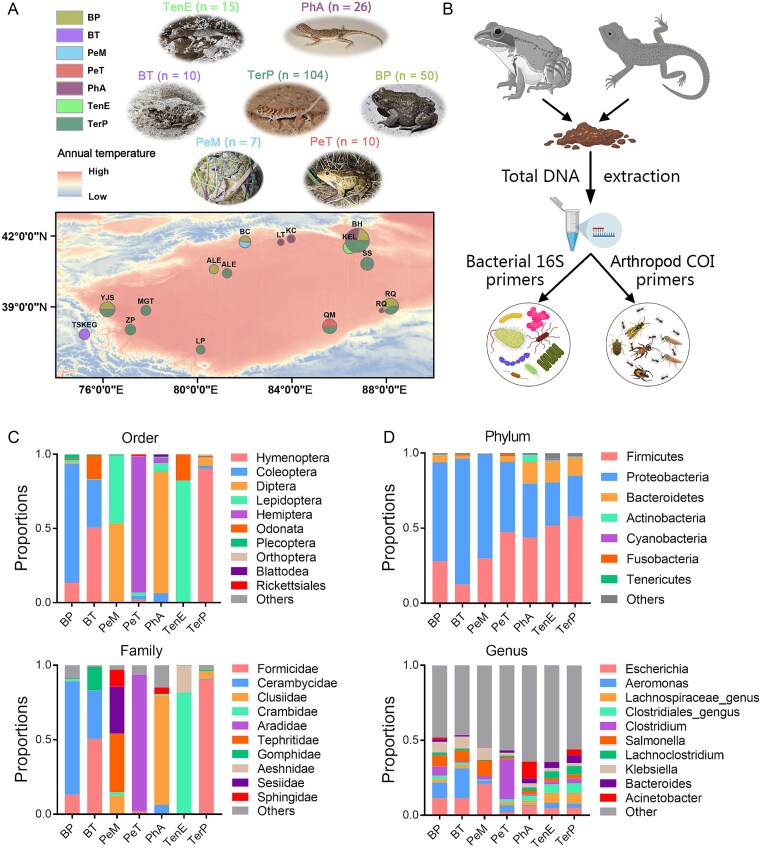
Experimental design and sample information. (a) Collection sites and typical morphology of the studied species. BP, *Bufotes pewzowi*; BT, *Bufotes taxkorensis*; PeM, *Pelophylax mongolius*; PeT, *Pelophylax terentievi*; PhA, *Phrynocephalus axillaris*; TenE, *Tenuidactylus elongatus*; TerP, *Teratoscincus przewalskii*. (b) Experimental design. The total environmental DNA extracted from the gut content were divided into two parts for bacterial 16S and insect CO I barcoding respectively. (c) Arthropod compositions in the samples at order and family levels. The mean values of each species were presented. (d) Bacterial compositions in the samples at phylum and genus levels. The mean values of each species were presented.

Samples were collected along the Tarim River during June and July 2024 (Fig. 1a). The study area (75.2162−88.1724 E, 37.1919−42.0170 N) spans 1000 km, representing the largest distance between collection sites (Supplementary Table S1). A total of 222 gut microbial samples were collected from 20 sampling sites (Fig. 1b−d), encompassing four amphibians (i.e. *B. pewzowi*, *B. taxkorensis*, *Pe. terentievi*, and *Pe. mongolius*) and three reptiles (i.e. *Ter. przewalskii*, *Ph. axillaris*, and *Ten. elongatus*) (detailed in Supplementary Data 1). After euthanized with MS-222 or ketamine, the animals were dissected with sterile tweezers to collect colon and rectum contents, which were mixed and transferred to 2 ml aseptic centrifuge tubes with 1.5 ml DNA storage solution (Novogene Co., Ltd). New gloves were used for sample collection of each individual to avoid cross contamination. All animal protocols in this study were reviewed and approved by the Animal Ethical and Welfare Committee of Chengdu Institute of Biology, Chinese Academy of Sciences (permit number: CIBDWLL2023201), in compliance with the ARRIVE guidelines 2.0 [38] and Guide for the Care and Use of Laboratory Animals (8th edition) published by National Research Council (US) Committee for the Update of the Guide for the Care and Use of Laboratory Animals [39].

### Arthropod COI amplicon sequencing (barcoding)

Cytochrome c oxidase subunit I (COI) mitochondrial gene fragments were performed to study arthropod community composition and diversity. Genomic DNA (gDNA) of the gut content was extracted using the Zymo Research BIOMICS DNA Microprep Kit, and its integrity was assessed via agarose gel electrophoresis. DNA concentration was measured using the Tecan F200 with PicoGreen staining. The COI gene fragments were amplified using specific primers (BF3: CCHGAYATRGCHTTYCCHCG and BR2: TCDGGRTGNCCRAARAAYCA) [34, 40, 41] with KOD-Plus-Neo DNA Polymerase. Polymerase chain reaction (PCR) products were verified through 2% agarose gel electrophoresis, purified using the Zymoclean Gel Recovery Kit, and quantified with the Qubit 2.0 Fluorometer. Libraries were prepared using the NEBNext Ultra II DNA Library Prep Kit and sequenced on the Illumina NovaSeq 6000 platform (PE250).

Data processing involved merging paired-end reads with FLASH, demultiplexing, and quality filtering using Trimmomatic [42] to remove low-quality and short reads. Sequence denoising and chimera removal were performed with DADA2 [43] in QIIME2, generating amplicon sequence variants. Taxonomic annotation was conducted using a Naïve Bayes classifier against the COins database [44]. Community composition analysis and diversity metrics (alpha and beta diversity) were calculated using R packages such as Vegan and Picante. Ordination and clustering analyses, including principal coordinate analysis (PCoA) and non-metric multidimensional scaling (NMDS), were performed to visualize community differences.

### 16S rRNA gene amplicon sequencing

To investigate the microbial community composition and diversity, full-length 16S ribosomal RNA (rRNA) mitochondrial gene amplicon sequencing was conducted using nanopore single-molecule sequencing technology. The gDNA samples were the same as those used for arthropod COI sequencing. To amplify the full-length 16S rRNA gene, primers 8F (5′-AGAGTTTGATCATGGCTCAG-3′) and 1492R (5′-CGGTTACCTTGTTACGACTT-3′) were used. PCR amplification was performed with MegaFi Fidelity DNA Polymerase (Applied Biological Materials Inc., G896) on an Applied Biosystems® PCR System 9700. The PCR reaction mixture (25 μL total volume) consisted of 5 μL of 5× MegaFi Buffer, 0.5 μL of 10 mM dNTPs, 1 μL of each primer (8F and 1492R), 0.5 μL of MegaFi DNA polymerase, 2 μL of template DNA (10 ng/μL), and 15 μL of nuclease-free water. The PCR cycling conditions were as follows: initial denaturation at 98°C for 30 s, followed by 25–30 cycles of denaturation at 98°C for 5 s, annealing at 54°C for 15 s, and extension at 72°C for 45 s, with a final extension at 72°C for 2 minutes. Each sample was amplified in triplicate to ensure reproducibility, and PCR products were pooled to minimize amplification bias. The amplified products were verified by electrophoresis on a 1% agarose gel, purified using the Zymoclean Gel Recovery Kit (D4008), and quantified with the Tecan F200. Equal molar amounts of purified PCR products were pooled for subsequent library preparation using the CycloneSEQ Universal Library Preparation Kit (H940-000013).

High-throughput sequencing was performed on the CycloneSEQ platform using the WuTong (WT) sequencing chip (H930-000001-00) (BGI, Shenzhen, China). The raw data were processed by basecalling to convert electrical signals into fastq-formatted sequences. Quality control was carried out using NanoFilt (v2.7.1) to remove low-quality reads (average quality score < 10) and length outliers (sequences shorter than 1400 bp or longer than 1600 bp) [45]. Potential chimeras were identified and removed using the Uchime algorithm with the gold database [46], resulting in high-quality, non-chimeric reads. Taxonomic annotation of the cleaned sequences was performed using Kraken2 [47], generating operational taxonomic unit (OTU) tables. Representative sequences for each OTU were aligned with MUSCLE, and a phylogenetic tree was constructed using FastTree [48]. The SILVA database (v138) was used for taxonomic assignment [49]. Alpha diversity indices (e.g. PD-whole-tree, Shannon, and Simpson) were calculated using the R packages *picante* and *vegan* [50].

**Table 1:** Arthropod abundance at order level.^a^

**Species**	**BP** (*n* = 50)	**BT** (*n* = 10)	**PeM** (*n* = 7)	**PeT** (*n* = 10)	**PhA** (*n* = 26)	**TenE** (*n* = 15)	**TerP** (*n* = 104)
**Blattodea**	0.02 ± 0.01^b^	0.01 ± 0.01^b^	0 ± 0^b^	0.14 ± 0.02^a^	1.75 ± 1.73^b^	0.02 ± 0.02^b^	0 ± 0^b^
**Coleoptera**	**79.92 ± 4.57** ^**a**^	**32.36 ± 13.2** ^**ab**^	0.19 ± 0.07^b^	2.55 ± 2.37^b^	5.72 ± 4.11^b^	0.25 ± 0.23^b^	2.03 ± 0.62^b^
**Diptera**	0.88 ± 0.34^ab^	0.25 ± 0.11^ab^	**52.78 ± 18.3** ^**ab**^	0.14 ± 0.06^ab^	**82.06 ± 6** ^**a**^	0.05 ± 0.04^b^	5.13 ± 1.91^b^
**Hemiptera**	0.38 ± 0.19^b^	0.01 ± 0.01^b^	0.13 ± 0.11^b^	**91.79 ± 3.69** ^**a**^	3.64 ± 1.59^b^	0.07 ± 0.05^b^	0.28 ± 0.15^b^
**Hymenoptera**	**13.56 ± 4.04** ^**ab**^	**50.61 ± 16.24** ^**ab**^	0.39 ± 0.06^ab^	2.12 ± 1.81^ab^	0.54 ± 0.28^b^	0.08 ± 0.04^b^	**90.23 ± 2.34** ^**a**^
**Lepidoptera**	1.69 ± 0.77^b^	0.16 ± 0.08^b^	**45.7 ± 18.59** ^**ab**^	2.05 ± 1.79^ab^	5.64 ± 3.49^ab^	**81.89 ± 9.11** ^**a**^	0.28 ± 0.13^b^
**Odonata**	0.56 ± 0.13^ab^	**15.99 ± 9.3** ^**b**^	0.04 ± 0.03^ab^	0.03 ± 0.01^ab^	0.12 ± 0.04^ab^	**17.59 ± 9.12** ^**b**^	1.01 ± 0.67^a^
**Orthoptera**	0.03 ± 0.01	0.02 ± 0.02	0.03 ± 0.03	0 ± 0	0.02 ± 0.01	0.01 ± 0	0.88 ± 0.87
**Plecoptera**	2.77 ± 1.62^a^	0.55 ± 0.2^a^	0.69 ± 0.54^ab^	0.06 ± 0.03^ab^	0.5 ± 0.28^ab^	0 ± 0^b^	0.14 ± 0.09^ab^
**Rickettsiales**	0.05 ± 0.03	0.03 ± 0.03	0 ± 0	1.09 ± 1.09	0 ± 0	0.03 ± 0.03	0.01 ± 0.01

a BP, *Bufotes pewzowi*; BT, *Bufotes taxkorensis*; PeM, *Pelophylax mongolius*; PeT, *Pelophylax terentievi*; PhA, *Phrynocephalus axillaris*; TenE, *Tenuidactylus elongatus*; TerP, *Teratoscincus przewalskii*. The data are presented as mean ± SE, and the interspecies differences are examined using Kruskal–Wallis tests and Bonferroni–Dunn tests. Different letters denote significant differences between groups (at threshold of *P* < .05). Values with abundance >10% are shown in bold.

### Bioinformatic and statistical analyses

Basic statistical analyses were performed using IBM SPSS v21.0 (IBM, Armonk, NY, USA) and R [51]. Alpha diversity of dietary arthropods and gut microbiota was assessed using the Shannon and PD-whole-tree indices. Differences in alpha diversity across animal classes were examined using a linear mixed model, with animal class as a fixed factor and species as a random factor. Interspecies variations were evaluated using the Kruskal–Wallis test, followed by post hoc analysis. Beta diversity was assessed using various distance matrices (e.g. Manhattan, Bray–Curtis, and Cao) for both dietary arthropods and gut microbiota. Among these, Bray–Curtis distance showed strong performance in explaining the total variance in dietary arthropod and gut microbial beta-diversity through the first two PCoA coordinates (Supplementary Tables S2 and S3), while Cao distance most effectively disperses the dietary arthropod samples, clearly separating samples from different species (Supplementary Figs. S1 and S2). These two distances were thus used for further analyses. The discriminative ability of each distance matrix to differentiate between species was evaluated using PCoA, and permutational multivariate analysis of variance (PERMANOVA, vegan package) [50], based on the proportion of variance explained by the first two coordinates and the statistical significance of interspecies differences. Among the tested distances, Bray–Curtis was identified as the most informative. Additionally, the Cao distance was used to assess dietary arthropod diversity, as it shares similarities with Bray–Curtis but places greater emphasis on sparsity, offering a more balanced representation of shared and sparse data. PCoA and NMDS were used to visualize the clustering of samples with animal class and species. Potential associations between the beta diversity of dietary arthropods and gut microbiota were evaluated using Mantel tests and Procrustes analyses (vegan package). Differential analyses were conducted using the Kruskal–Wallis test (to assess variations among animal species), linear mixed models (to evaluate variations between animal classes), and linear discriminant analysis Effect Size (LEfSe, to analyze class-level variations) [52]. Pairwise associations between the relative abundance of arthropod and microbial taxa were examined using Spearman correlation. *P*-values were adjusted using the Benjamini–Hochberg (BH) correction method. All graphical representations were created using GraphPad Prism 5 and ggplot2 [53].

## Results

### Dietary arthropod and gut microbial composition

The predominant dietary insects vary notably among species: *B. pewzowi* primarily consumes Coleoptera (Cerambycidae); *B. taxkorensis* mainly consumes Hymenoptera (Formicidae) and Coleoptera (Cerambycidae); *Pe. mongolius* feeds on Diptera (Tephritidae) and Lepidoptera (Sesiidae); *Pe. terentievi* on Hemiptera (Aradidae); *Ph. axillaris* on Diptera (Clusiidae); *Ten. elongatus* on Lepidoptera (Crambidae); and *Ter. przewalskii* on Hymenoptera (Formicidae) (Fig. 1c, Tables 1 and 2). The composition of dietary insects differs markedly among species, even between closely related species within the same genus (e.g. *Pe. mongolius* and *Pe. terentievi*). In contrast, the gut microbial compositions of these species are convergent at the phylum level, with Firmicutes, Proteobacteria, and Bacteroidetes being predominant across all species (Fig. 1d, Table 3). At the genus level, the most abundant bacteria (>5%) include *Escherichia*, *Aeromonas*, *Clostridium*, *Salmonella*, and *Klebsiella* in *B. pewzowi*; *Escherichia*, *Aeromonas*, *Salmonella*, and *Klebsiella* in *B. taxkorensis*; *Escherichia*, *Salmonella*, and *Klebsiella* in *Pe. mongolius*; *Clostridium* in *Pe. terentievi*; *Acinetobacter* and *Escherichia* in *Ph. axillaris*; an unclassified *Clostridium* genus and an unclassified *Lachnospiraceae* genus in *T. elongatus*; and *Lachnoclostridium*, *Escherichia*, *Bacteroides*, as well as unclassified genera of *Clostridium* and *Lachnospiraceae* in *Ter. przewalskii* (Fig. 1d, Table 4).

**Table 2:** Arthropod abundance at family level.^a^

**Species**	**BP** (*n* = 50)	**BT** (*n* = 10)	**PeM** (*n* = 7)	**PeT** (*n* = 10)	**PhA** (*n* = 26)	**TenE** (*n* = 15)	**TerP** (*n* = 104)
**Aeshnidae**	0.03 ± 0.02^b^	0 ± 0^b^	0 ± 0^b^	0.01 ± 0.01^b^	0.01 ± 0^b^	**17.58 ± 9.11** ^**a**^	0 ± 0^b^
**Aradidae**	0 ± 0^b^	0 ± 0^b^	0 ± 0^b^	**91.66 ± 3.69** ^**a**^	0 ± 0^b^	0.02 ± 0.01^b^	0 ± 0^b^
**Cerambycidae**	**76.04 ± 4.75** ^**a**^	**32.35 ± 13.2** ^**ab**^	0.04 ± 0.03^ab^	0.01 ± 0.01^b^	5.69 ± 4.11^ab^	0.01 ± 0.01^b^	0.63 ± 0.4^ab^
**Clusiidae**	0.04 ± 0.01^b^	0 ± 0^b^	**12.21 ± 12.02** ^**a**^	0 ± 0^b^	**73.5 ± 7.6** ^**a**^	0.04 ± 0.03^b^	5.07 ± 1.91^b^
**Crambidae**	1.38 ± 0.76^ab^	0.08 ± 0.05^ab^	2.18 ± 2.11^ab^	0.12 ± 0.03^ab^	0.95 ± 0.71^ab^	**81.56 ± 9.06** ^**a**^	0.21 ± 0.13^b^
**Formicidae**	**13.2 ± 4.05** ^**ab**^	**50.61 ± 16.24** ^**ab**^	0.31 ± 0.07^ab^	2.03 ± 1.82^ab^	0.5 ± 0.28^b^	0.07 ± 0.03^b^	**90.22 ± 2.34** ^**a**^
**Gomphidae**	0.23 ± 0.08^b^	**15.98 ± 9.3** ^**a**^	0.01 ± 0.01^ab^	0 ± 0^b^	0.01 ± 0.01^b^	0 ± 0^b^	0.92 ± 0.67^b^
**Sesiidae**	0.03 ± 0.01^b^	0.03 ± 0.02^b^	**31.3 ± 17.56** ^**a**^	0 ± 0^b^	0.01 ± 0^b^	0 ± 0^b^	0.01 ± 0^b^
**Sphingidae**	0 ± 0^b^	0.01 ± 0.01^ab^	**11.62 ± 11.62** ^**ab**^	0 ± 0^b^	4.49 ± 3.26^a^	0 ± 0^b^	0 ± 0^b^
**Tephritidae**	0.01 ± 0^b^	0.01 ± 0.01^b^	**39.42 ± 18.59** ^**a**^	0 ± 0^b^	0 ± 0^b^	0 ± 0^b^	0 ± 0^b^

a BP, *Bufotes pewzowi*; BT, *Bufotes taxkorensis*; PeM, *Pelophylax mongolius*; PeT, *Pelophylax terentievi*; PhA, *Phrynocephalus axillaris*; TenE, *Tenuidactylus elongatus*; TerP, *Teratoscincus przewalskii*. The data are presented as mean ± SE, and the interspecies differences are examined using Kruskal–Wallis tests and Bonferroni–Dunn tests. Different letters denote significant differences between groups (at threshold of *P* < .05). Values with abundance >10% are shown in bold.

**Table 3:** Gut bacterial abundance at phylum level.^a^

**Species**	**BP** (*n* = 50)	**BT** (*n* = 10)	**PeM** (*n* = 7)	**PeT** (*n* = 10)	**PhA** (*n* = 26)	**TenE** (*n* = 15)	**TerP** (*n* = 104)
**Actinobacteria**	0.21 ± 0.03^ab^	0.13 ± 0.04^ab^	0.06 ± 0.02^b^	0.13 ± 0.05^ab^	4.45 ± 2.26^ab^	0.58 ± 0.11^a^	0.42 ± 0.03^a^
**Bacteroidetes**	4.58 ± 1.29^ab^	1.89 ± 1.1^ab^	0.25 ± 0.17^b^	3.77 ± 2.49^ab^	**14.35 ± 3.61** ^**ab**^	**14.18 ± 2.32** ^**a**^	**12.09 ± 1.17** ^**ab**^
**Cyanobacteria**	0.05 ± 0.02^b^	0.02 ± 0.01^b^	0 ± 0^b^	0.03 ± 0.01^b^	0.17 ± 0.05^ab^	0.88 ± 0.28^a^	0.31 ± 0.08^ab^
**Firmicutes**	**27.94 ± 4.46** ^**b**^	**12.45 ± 5.97** ^**b**^	**29.85 ± 14.67** ^**ab**^	**47.41 ± 8.25** ^**ab**^	**43.73 ± 5.85** ^**ab**^	**51.84 ± 6.86** ^**ab**^	**57.63 ± 2.84** ^**a**^
**Fusobacteria**	0.19 ± 0.07^ab^	0.89 ± 0.3^a^	0 ± 0^ab^	1.5 ± 1.18^a^	0.01 ± 0^b^	0.01 ± 0^ab^	0.02 ± 0^ab^
**Proteobacteria**	**65.8 ± 4.94** ^**a**^	**83.79 ± 7.17** ^**a**^	**69.53 ± 14.74** ^**ab**^	**46.5 ± 9.16** ^**ab**^	**35.98 ± 7.28** ^**ab**^	**28.58 ± 7.32** ^**ab**^	**27.31 ± 3.28** ^**b**^
**Tenericutes**	0.23 ± 0.1^b^	0.03 ± 0.01^b^	0.01 ± 0^b^	0.06 ± 0.02^ab^	0.07 ± 0.02^b^	0.38 ± 0.1^a^	0.14 ± 0.01^ab^

a BP, *Bufotes pewzowi*; BT, *Bufotes taxkorensis*; PeM, *Pelophylax mongolius*; PeT, *Pelophylax terentievi*; PhA, *Phrynocephalus axillaris*; TenE, *Tenuidactylus elongatus*; TerP, *Teratoscincus przewalskii*. The data are presented as mean ± SE, and the interspecies differences are examined using Kruskal–Wallis tests and Bonferroni–Dunn tests. Different letters denote significant differences between groups (at threshold of *P* < .05). Values with abundance >10% are shown in bold.

**Table 4:** Gut bacterial abundance at genus level.^a^

**Species**	**BP** (*n* = 50)	**BT** (*n* = 10)	**PeM** (*n* = 7)	**PeT** (*n* = 10)	**PhA** (*n* = 26)	**TenE** (*n* = 15)	**TerP** (*n* = 104)
***Acinetobacter***	1.29 ± 0.62^ab^	0.05 ± 0.02^ab^	0.07 ± 0.02^ab^	0.02 ± 0.01^b^	**11.16 ± 5.16** ^**ab**^	0.29 ± 0.11^a^	3.98 ± 1.2^ab^
***Aeromonas***	**10.43 ± 2.38** ^**a**^	**19.88 ± 7.55** ^**ab**^	2.33 ± 1.81^ab^	4.52 ± 3.72^ab^	0.73 ± 0.59^b^	3.36 ± 2.33^ab^	2.73 ± 0.85^ab^
***Bacteroides***	1.62 ± 0.42^ab^	1.06 ± 0.62^ab^	0.09 ± 0.07^b^	1.5 ± 1.2^ab^	3.5 ± 1.32^ab^	3.94 ± 0.76^a^	5.15 ± 0.69^a^
***Clostridium***	6.16 ± 2.78^b^	0.8 ± 0.57^b^	2.42 ± 1.52^ab^	**26.73 ± 9.55** ^**a**^	0.79 ± 0.15^b^	1.8 ± 0.32^ab^	2.88 ± 0.35^ab^
***Escherichia***	**11.39 ± 1.26** ^**b**^	**11.44 ± 2.81** ^**ab**^	**20.84 ± 7.18** ^**a**^	2.14 ± 0.85^ab^	5.96 ± 1.85^ab^	4.7 ± 2.09^ab^	5.14 ± 0.87^b^
***Klebsiella***	6.99 ± 0.73^a^	7.88 ± 1.61^ab^	8.23 ± 2.19^ab^	1.83 ± 0.58^ab^	2.74 ± 0.89^b^	2.18 ± 1.2^ab^	2.05 ± 0.32^b^
***Lachnoclostridium***	1.97 ± 0.74^ab^	1.44 ± 0.76^ab^	0.35 ± 0.32^b^	1.15 ± 0.28^ab^	1.97 ± 0.55^ab^	4.6 ± 1.4^ab^	5.65 ± 0.41^a^
***Salmonella***	7.46 ± 0.87^a^	7.2 ± 1.42^ab^	**10.24 ± 2.75** ^**ab**^	1.35 ± 0.37^ab^	2.76 ± 0.86^b^	2.27 ± 1.05^ab^	2.76 ± 0.44^b^

a BP, *Bufotes pewzowi*; BT, *Bufotes taxkorensis*; PeM, *Pelophylax mongolius*; PeT, *Pelophylax terentievi*; PhA, *Phrynocephalus axillaris*; TenE, *Tenuidactylus elongatus*; TerP, *Teratoscincus przewalskii*. The data are presented as mean ± SE, and the interspecies differences are examined using Kruskal–Wallis tests and Bonferroni–Dunn tests. Different letters denote significant differences between groups (at threshold of *P* < .05). Values with abundance >10% are shown in bold.

**Figure 2:**
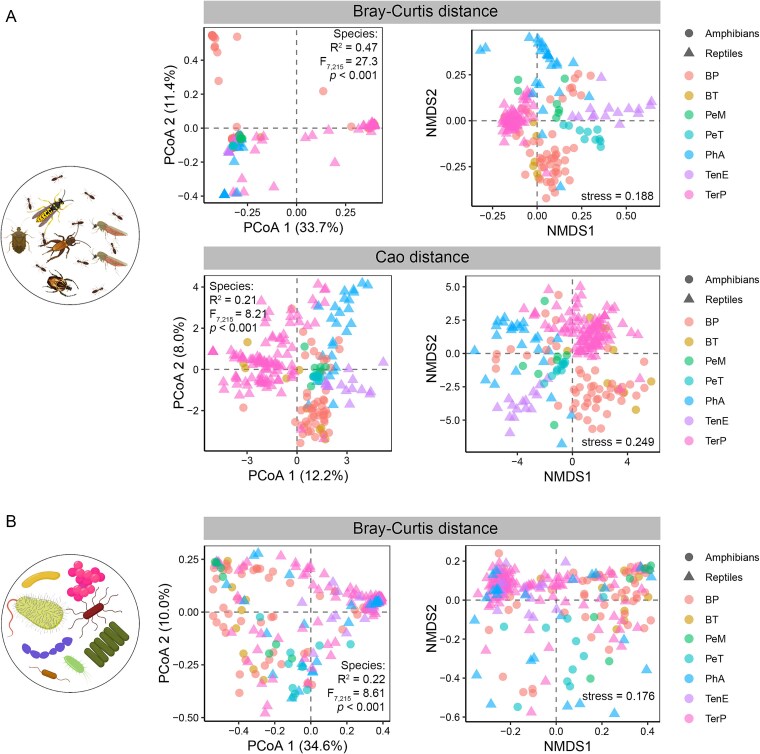
Variations in beta-diversity of gut arthropods (a) and bacteria (b). PCoA and NMDS analyses were performed to visualize the clustering of samples based on species. PERMANOVA was conducted to assess the influence of species on the arthropod and bacterial beta-diversity, with the statistical results displayed in the PCoA scatter plot. Bray–Curtis distances were calculated to represent beta-diversity for both arthropod and bacterial communities. Additionally, Cao distance was considered for arthropod data, as it provides the best performance in distinguishing samples from different species (Supplementary Figs. S1 and S2). More detailed statistical results, including PCoA and PERMANOVA outcomes for other distance metrics, are provided in Supplementary Tables S1 and S2.

**Figure 3:**
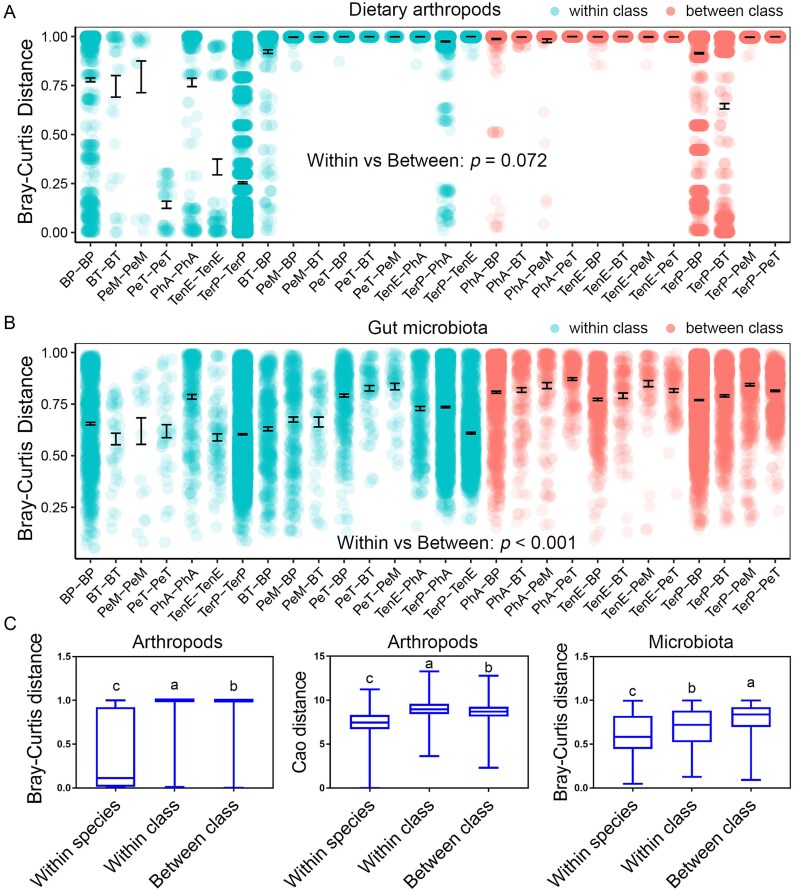
Dietary arthropod and gut microbial similarity across taxonomic levels. (a and b) Dot plots illustrating dietary arthropod (a) and gut microbial (b) similarity, measured as pairwise Bray–Curtis distances between samples for each species pairs. A linear mixed model was used to evaluate differences in similarity between pairs within the same animal class and pairs between different animal classes. The classification of species pairs as belonging to the same or different animal classes was treated as a fixed factor, while the specific species pairs were treated as a random factor. (c) Box plots displaying dietary arthropod and gut microbial similarity (Bray–Curtis or Cao distances) for sample pairs at different taxonomic levels: Within the same species, within the same class but different species, and between different classes. The data were analyzed using one-way ANOVA followed by an S-N-K post hoc test with random sample selection (sample size = 1000). Different letters indicate significant differences (*P* < .05) between groups.

Both dietary arthropod and gut microbial compositions vary significantly among species (Fig. 2, Supplementary Tables S1 and S2), indicating substantial differentiation in trophic niches and gut microbiota. However, they differ in their ability to reflect host class (Amphibia vs. Reptilia) (Fig. 3). The composition of dietary insects does not exhibit greater similarity among species within the same class than between the two classes (Fig. 3a and c). Although LEfSe analysis identifies certain arthropod taxa (e.g. Hymenoptera in reptiles and Hemiptera in amphibians) as characteristic of either amphibians or reptiles (Supplementary Fig. S3a), these patterns are not statistically significant after accounting for interspecific variation using a mixed linear model with species as a random factor. In contrast, gut microbial composition is more strongly associated with host phylogeny than with dietary composition, showing greater similarity within the same class than between classes (Fig. 3b and c). Specifically, amphibians tend to harbor higher proportions of Proteobacteria (*P* = .087), lower proportions of Firmicutes (*P* = .054), a higher Firmicutes-to-Bacteroidetes (F/B) ratio (*P* = .099), and a higher abundance of *Klebsiella pneumoniae* (*P* = .074). Moreover, the relative abundance of Bacteroidetes is significantly higher in amphibians than in reptiles (*P* < .001), including elevated levels of the genera *Bacteroides* (*P* < .001) and *Blautia* (*P* = .003), as well as the species *Odoribacter splanchnicus* (*P* = .015) (Supplementary Fig. S3b–d).

In addition to species, we also examined the influences of a physiological factor (snout–vent length, SVL) and an environmental factor (sympatry vs. allopatry) on dietary arthropod and gut microbial compositions. SVL showed no significant association with dietary arthropod composition at either the across-species or within-species level (PERMANOVA). In contrast, SVL was significantly associated with gut microbial composition, both across species and within *B. pewzowi* and *B. taxkorensis* (Supplementary Fig. S4a–c), indicating a relative independence between dietary arthropod and gut microbiota variation. For *Ph. axillaris* and *Te. przewalskii*, which include both sympatric and allopatric populations in our dataset, we found that sympatric populations were more similar than allopatric populations in both dietary arthropod and gut microbial compositions (Supplementary Fig. S4d and e), suggesting potential interspecific competition.

### Associations in diversity between dietary arthropod and gut microbiota

**Figure 4:**
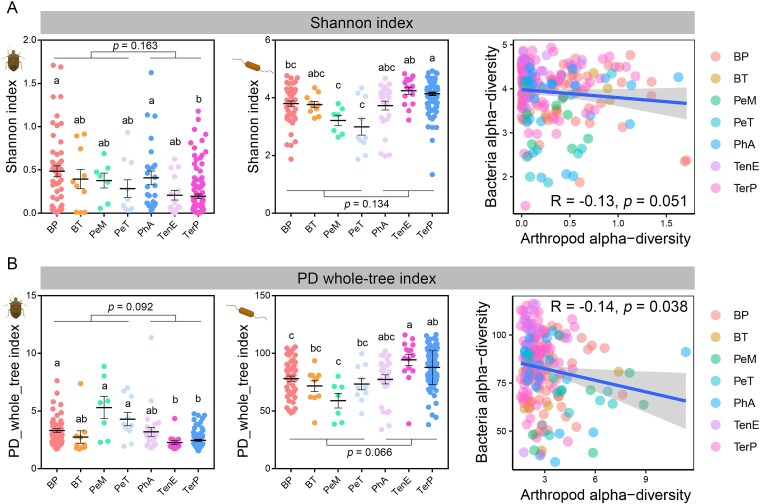
Variations in alpha-diversity of gut arthropods and bacteria. (a and b) The Shannon (a) and PD whole-tree (b) indices were calculated to present the alpha-diversity. Kruskal–Wallis tests were performed to evaluate the variations in alpha-diversity indices (mean ± SE) across species, and different letters denote significant differences (*P* < .05 in post hoc test after BH corrections) between species. The associations between gut arthropod and bacterial diversity were examined with Spearman correlations. Each dot denotes the value of each sample. The confidence interval level of the smooth line is 0.95.

The alpha-diversity of both dietary arthropods and gut microbiota, measured by Shannon and PD_whole_tree indices, varies significantly among species (Fig. 4). Higher dietary alpha-diversity is observed in *B. pewzowi* and *Pe. mongolius*, whereas *Ter. przewalskii* shows the lowest values. In contrast, the highest gut microbial alpha-diversity is found in *Ter. przewalskii* and *Ten. elongatus*, while *Pe. mongolius* exhibits the lowest. No significant differences in alpha-diversity indices are detected between amphibian and reptile species (*P* > .05). When considering all samples, marginally significant negative correlations are observed between dietary arthropod and gut microbial alpha-diversity indices (Shannon: *R* = −0.13, *P* = .051; PD_whole_tree: *R* = −0.14, *P* = .038; Fig. 4). However, these correlations are not significant when analyzed within individual species or within the same animal class.

**Figure 5:**
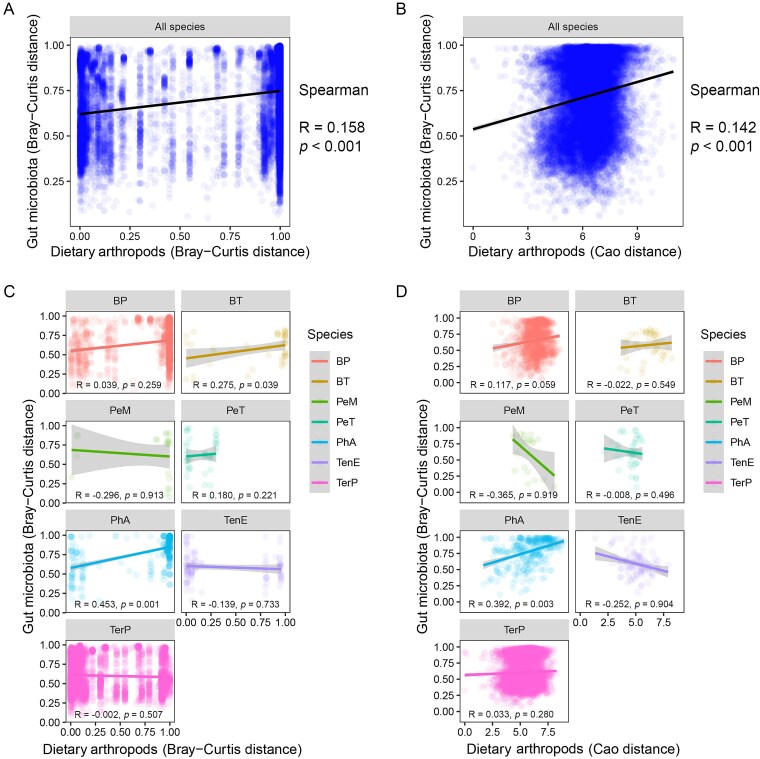
Associations between gut arthropod and bacterial composition. (a and b) Correlations between gut arthropod (Cao or Bray–Curtis distances) and bacterial (Bray–Curtis distance) beta-diversity across species. (c and d) Correlations between gut arthropod (Cao or Bray–Curtis distances) and bacterial (Bray–Curtis distance) beta-diversity for each species. *Mantel* tests were performed to examine the significance of the associations. The confidence interval level of the smooth line is 0.95.

Regarding beta-diversity, since both dietary arthropod and gut microbial beta-diversity vary with species and class, correlation analyses were performed at multiple taxonomic levels. Across all samples, significant positive correlations are detected (*P* < .001, Mantel tests; Fig. 5a and b and Supplementary  Fig.  S5), and these correlations remain significant within each animal class (Supplementary Figs. S6 and S7). However, at the species level, significant correlations (*P* < .05) are observed only in *Ph. axillaris* (Fig. 5c and d and Supplementary  Fig.  S8).

### Interactions between dietary arthropods and gut microbes

**Figure 6:**
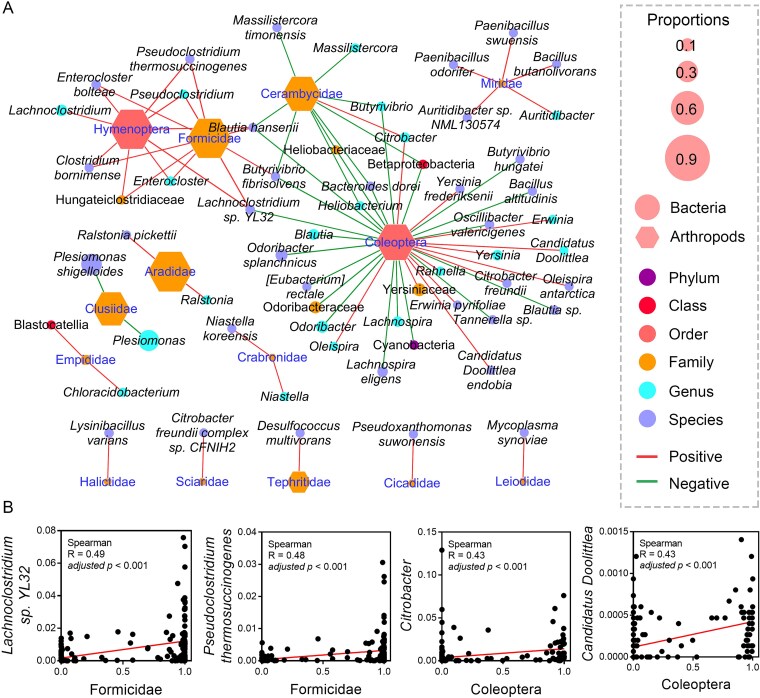
Network displaying the significant pairwise correlations between arthropod and bacterial taxa across all the amphibian and reptile species. Only the correlations meeting *P* < .001 (Spearman and BH correction) and |*R*| > 0.4 were presented. (b) Pairwise correlations showing the highest |*R*| values. The lines denote the linear fitting of the data.

Correlation analyses were performed between the relative abundances of individual arthropod and gut microbial taxa. When considering all samples, Coleoptera, Hymenoptera, Formicidae, and Cerambycidae were identified as key taxa due to their high relative abundance and central roles in the correlation network (|*R*| > 0.4, adjusted *P* < .001, Spearman correlation; Fig. 6a). Notably, Hymenoptera and Formicidae exhibit primarily positive correlations with gut microbes, whereas Cerambycidae show predominantly negative correlations. Most gut microbes significantly correlated with dietary arthropods are at the genus or species level and have relatively low abundances. Among the potential arthropod-microbe interactions, the four most significant positive pairs are Formicidae–*Lachnoclostridium* sp. YL32, Formicidae–*Pseudoclostridium thermosuccinogenes*, Coleoptera–*Citrobacter*, and Coleoptera–*Candidatus Doolittlea* (Fig. 6b).

Correlations were also analyzed for each species (Supplementary Fig. S9). *Ph. axillaris* exhibited the highest number of significant pairwise correlations (|*R*| > 0.4, *P* < .001), underscoring the core roles of Lepidoptera, Diptera, Hymenoptera, Crambidae, and Clusiidae in the correlation networks. *B. pewzowi* had the second highest number of significant correlations, highlighting Hymenoptera, Coleoptera, Blattodea, Clusiidae, Culicidae, Gomphidae, Anthocoridae, Mycetophilidae, Termitidae, and Hesperiidae. Several core arthropod taxa, such as Clusiidae and Gomphidae, were shared across the arthropod-microbe networks of different species (Supplementary Fig. S10).

## Discussion

### Variations of diet and gut microbiota with host phylogeny

The relationship between phylogeny and diet is a central focus in evolutionary biology. Closely related taxa often share cranial morphology and digestive physiology that facilitate similar dietary patterns [54–56], and phylogeny is considered as an important constraint on the food-web structure [57]. However, despite significant interspecific differences in dietary preferences, these differences did not align with higher-level taxonomic distinctions in this study. In fact, dietary convergence can occur among distantly related species occupying similar ecological niches, and vice versa [20]. From the perspective of the study organisms, none of the species investigated here is dietary specialists; all are insectivorous and possess the ability to digest chitin. This suggests a higher likelihood that environmental pressures during the speciation process may have driven shifts in their original dietary habits. From an environmental standpoint, desert habitats are characterized by low niche diversity and high competition among sympatric species. Over time, such competitive pressure may have promoted divergent dietary strategies among phylogenetically related species sharing the same habitat. Thus, while phylogeny plays a role in shaping dietary patterns, ecological factors and adaptive evolution are also crucial drivers [55], contributing to dietary variability even among closely related species. Currently, studies investigating the relationship between phylogeny and diet at the community level remain scarce in amphibians and reptiles. Whether such a link exists, and how dietary preferences have evolved in these groups, warrants further exploration across a wider range of ecological contexts and more taxonomically diverse species.

**Figure 7:**
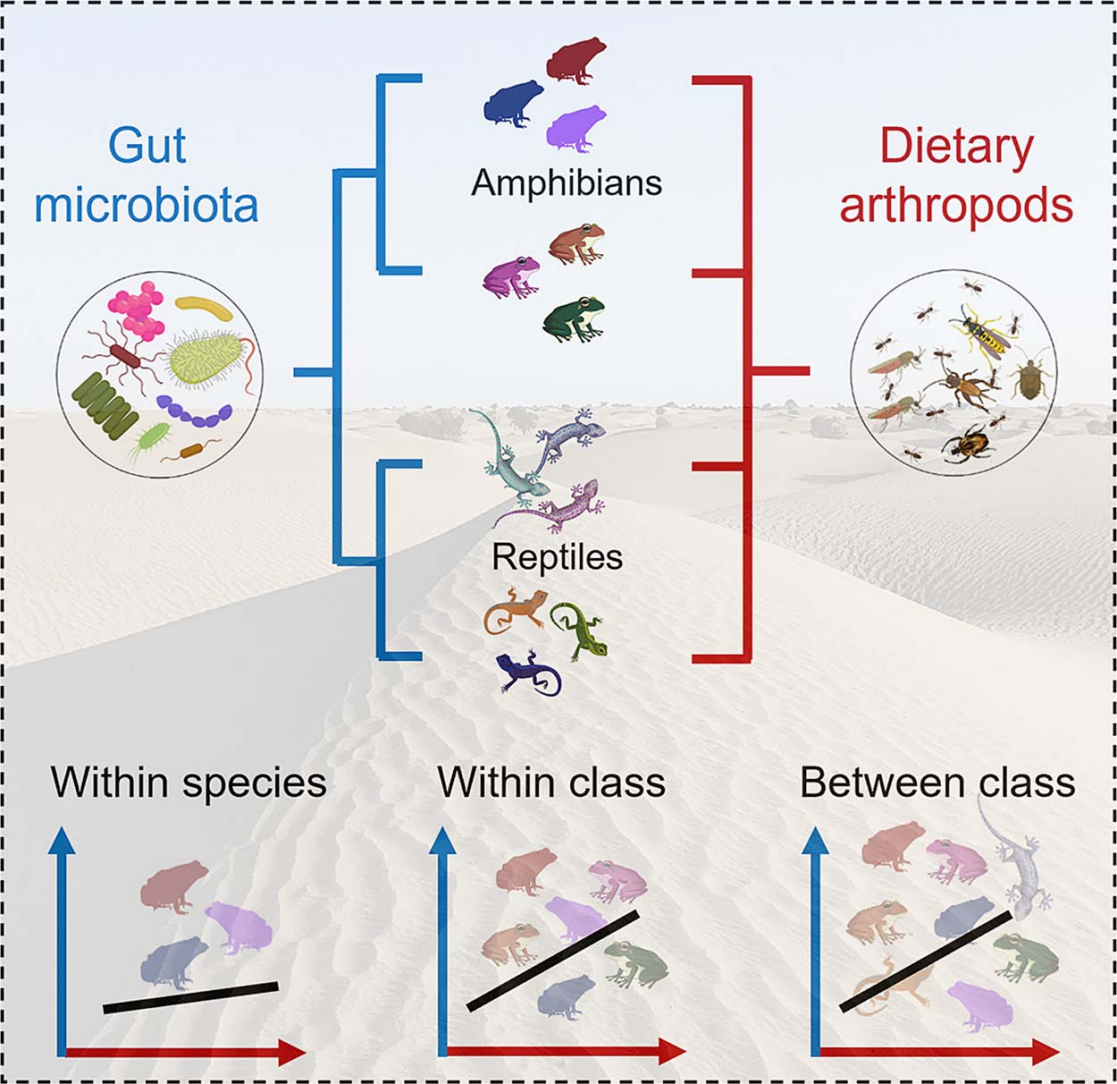
A summary of the major findings in this study. Dietary arthropods, unlike gut microbiota, do not reflect host phylogeny and thus do not mediate phylosymbiosis. The correlations between dietary arthropods and gut microbiota are not significant for samples from the same species (different populations), reflecting the resilience of gut microbiota to real-time dietary changes; however, the correlations are significant for samples across species, reflecting the significance of diet in shaping gut microbiota at species level.

Unlike dietary profiles, variation in gut microbial composition more closely reflects host phylogeny, suggesting the presence of phylosymbiosis among desert-dwelling amphibians and reptiles, as previously reported [37]. Phylosymbiosis may arise from host genetic or physiological traits that shape microbial communities [58], ecological filtering or environmental consistency within lineages [59, 60], and co-divergence or coevolution [61, 62]. In the present study, the observed phylosymbiotic patterns are at least partly attributable to similarities in ecological niches among species. Notably, the gut microbiota of amphibian species is dominated by Proteobacteria, which are more prevalent in aquatic environmental microbiota than in terrestrial habitats [63]. Given the more frequent contact between amphibians and aquatic environments, and the fact that waterborne microbial communities are a major source of gut microbiota in amphibians [63], the differences in gut microbiota between amphibians and reptiles may be explained by their contrasting lifestyles. These lifestyle differences, in turn, may contribute to the observed phylosymbiotic patterns. In combination, the results reject our first hypothesis that dietary composition may explain the phylosymbiosis observed in desert-dwelling amphibians and reptiles (Fig. 7).

### Variations of gut microbiota with diet

The influence of diet on gut microbiota has been widely demonstrated across interspecific or interpopulation comparisons [64], seasonal or long-term observations for the same populations [21, 22], and short-term controlled experiments within species [65]. Consistent with previous findings, our study also revealed a significant real-time correlation between diet and gut microbiota at the interspecific level. We suggest that this relationship is not a reflection of short-term microbial responses to diet, but rather the outcome of long-term adaptive processes shaped by host-diet-microbiota interactions. Such associations likely arise through evolutionary timescales, driven by dietary divergence across species [66]. Nutritional composition may act as a selective filter, shaping microbial communities over time [67, 68]. In parallel, host-mediated selection may reinforce these associations, as certain microbes enhance the host’s ability to digest and utilize specific diets [12], ultimately contributing to host fitness. Thus, at the interspecific level, the diet-microbiota correlation likely reflects species-specific adaptations and evolutionary filtering [69].

In contrast, no clear real-time association between diet and microbiota was observed at the intraspecific level, where no diet differentiation occurs. This may be due to functional redundancy and compositional stability within microbial communities [70], enabling them to buffer against fluctuations [30]. Rather than altering community composition, microbes may respond via gene transcriptional plasticity, broadening their ecological niche, and enhancing resilience to environmental variation [71, 72]. From the host’s perspective, such microbial plasticity is likely more advantageous than frequent community turnover, which could disrupt gut homeostasis and impose additional energetic costs. Importantly, the absence of diet-microbiota correlation within species may underscore the role of host fitness in shaping these relationships. Short-term dietary fluctuations may be insufficient to establish stable associations, as such relationships are more likely to emerge at the population or species level through selection favoring individuals with beneficial diet–microbiota configurations following divergence in diet composition or availability [73].

Our findings at the intraspecific level seem to be different from those of laboratory-based or controlled studies investigating the effects of diet on gut microbiota, where the variations of gut microbiota with dietary changes are rapid and significant [19]. This discrepancy may be attributed to several key differences between laboratory and wild individuals. In laboratory settings, diets are typically less complex, more stable, and lack the natural variability observed in the wild. Dietary changes in controlled experiments, or even seasonal variations in the field, can be abrupt, consistent, and sustained over longer periods, which may enhance the direct impact of diet and host-mediated selection on the gut microbiota. In contrast, wild individuals are exposed to more diverse and fluctuating diets, which may reduce the strength and consistency of dietary filtering effects. This is particularly true for the situation for our study which specifically focuses on the real-time and dynamic relationship between dietary composition and gut microbiota. Additionally, the gut microbial composition and diversity of lab-reared individuals can differ substantially from those of wild counterparts, potentially leading to differences in the functional redundancy and resilience of their microbial communities in response to dietary changes. It should also be noted that the miss of correlations at the whole similarity level does not mean the absence of the influences of certain nutrients in the diet on individual microbes and functional genes, especially those associated with host health [74, 75]. Currently, numerous questions remain unanswered, including the nature of diet–gut microbiota relationships across different taxonomic or ecological levels and the factors shaping these associations, all of which require further in-depth investigation.

Together, these findings highlight taxonomic-level differences in the diet–microbiota relationship (Fig. 7) and potentially support the view that hosts and their gut microbiota form an integrated evolutionary unit to natural selection.

### Conservation implications

Our results indicate significant dietary differentiation among desert-dwelling amphibian and reptile species. Notably, dietary arthropod diversity does not show a positive correlation with species population size. For instance, *B. pewzowi* and *Ter. przewalskii* have relatively large populations among amphibians and reptiles in the Tarim Desert, respectively, but neither exhibits higher dietary arthropod diversity compared to other species. This suggests that their adaptive success in desert environments may rely more on the abundance rather than the breadth of their prey. These findings imply that amphibians and reptiles in the Tarim Desert have developed relatively stable and independent dietary niches. As such, changes in local insect composition and abundance could lead to shifts in the community structure and population sizes of these vertebrates. This indicates that environmental and climatic changes may impact amphibians and reptiles not only directly, as these animals are highly sensitive to such changes, but also indirectly through trophic interactions. Given the inherently low biodiversity of desert ecosystems, their ecological networks (e.g. food webs) are more fragile, and organisms occupying higher trophic levels, such as amphibians and reptiles, may be more vulnerable to environmental perturbations. However, this lower network complexity may offer an advantage: it potentially facilitates the monitoring of species interactions and ecosystem dynamics. Therefore, future research should adopt a more integrative network-based approach to monitor biodiversity changes in the Tarim Desert and inform conservation strategies.

Compared to reptiles, amphibians show greater dependency on water bodies and are more vulnerable to drought conditions. Moreover, our findings reveal that amphibians harbor higher levels of potential pathogenic bacteria in their gut microbiota, including *Escherichia coli*, *Aeromonas* spp., *K. pneumoniae*, and *Salmonella enterica*, posing potential health risks. As discussed above, these bacteria, all belonging to Proteobacteria, are likely acquired from aquatic environments. In fact, not only microbial contaminants, but also pollutants and antibiotic resistance genes, can spread more readily through water bodies [76, 77]. At the same time, because water buffers the extreme temperature fluctuations of desert environments, aquatic microbial communities may survive longer. These factors suggest that amphibians in the Tarim Desert may be particularly susceptible to pollution and disease risks. Continuous monitoring of amphibian diversity and health in this region is therefore essential.

## Conclusion

This study explored variations in dietary arthropods and gut microbiota across seven desert-dwelling amphibian and reptile species to evaluate the influence of diet on gut microbiota in natural conditions. Results reveal distinct dietary arthropod compositions and gut microbial communities between species. Compared to dietary profiles, gut microbiota composition more closely reflects host phylogeny, supporting the presence of phylosymbiosis, which is likely driven by host evolutionary history or ecological adaptation rather than dietary divergence. We observed significant correlations between dietary composition and gut microbiota, implying long-term co-adaptation between hosts, their diets, and associated microbes. These findings highlight the complex interplay between ecological and evolutionary factors in shaping the trophic and microbial niches of desert vertebrates and underscore the importance of integrating dietary and microbial data in understanding host–microbe interactions.

## Supplementary Material

Supplementary_figures_R2_ycaf213

Supplementary_tables_ycaf213

## Data Availability

The sequencing data were deposited in the National Genomic Data Center (https://ngdc.cncb.ac.cn/) under accession number CRA026452 and CRA026380. The code and data used in the analyses are available in Figshare (https://doi.org/10.6084/m9.figshare.30264709.v1).
