# Supplementary material for: In-situ diet–microbiota associations across taxonomic scales in desert-dwelling amphibians and reptiles
Source: ISME Commun. 2025 Nov 18;5(1):ycaf213. doi: 10.1093/ismeco/ycaf213 (PMC12694432; doi:10.1093/ismeco/ycaf213)
Supplement: Supplementary_figures_R2_ycaf213 [file supplementary_figures_r2_ycaf213.docx]

**Supplementary figures**


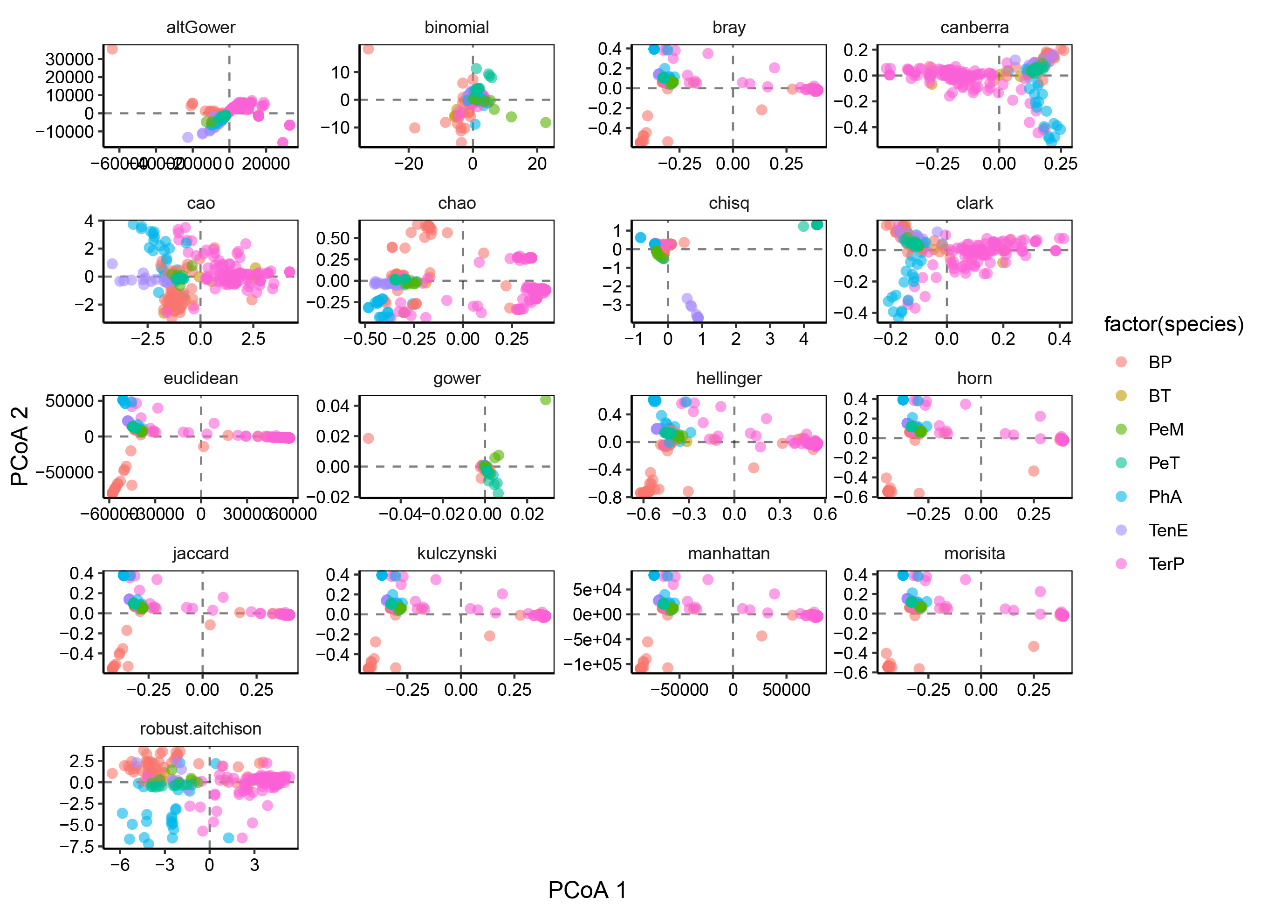


**Figure S1** PCoA scatter plots showing the clustering of arthropod samples based on species. Different distance matrices were calculated for the arthropod OTU table, and their performance on clustering the samples based on species were compared intuitively.


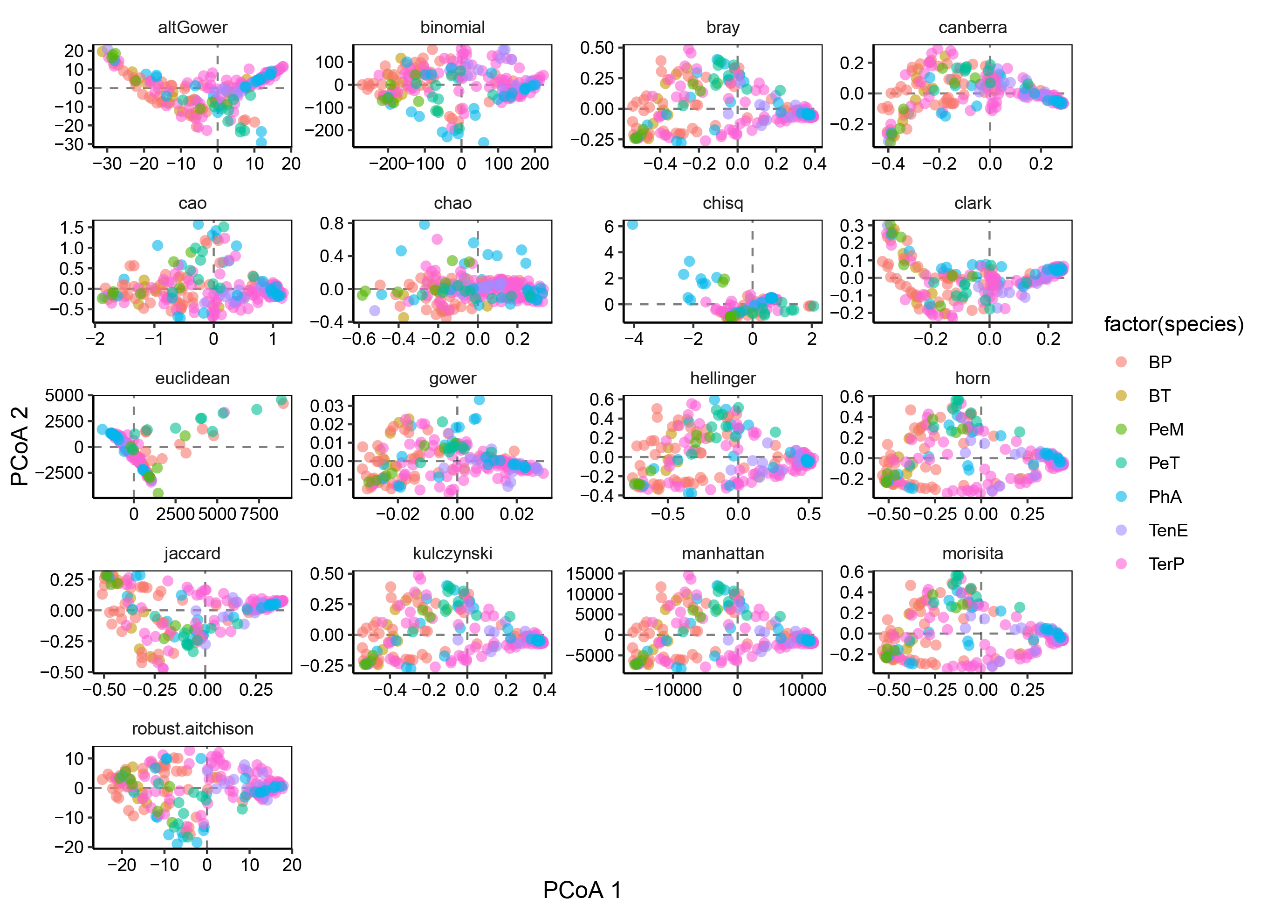


**Figure S2** PCoA scatter plots showing the clustering of bacterial samples based on species. Different distance matrices were calculated for the bacterial OTU table, and their performance on clustering the samples based on species were compared intuitively.


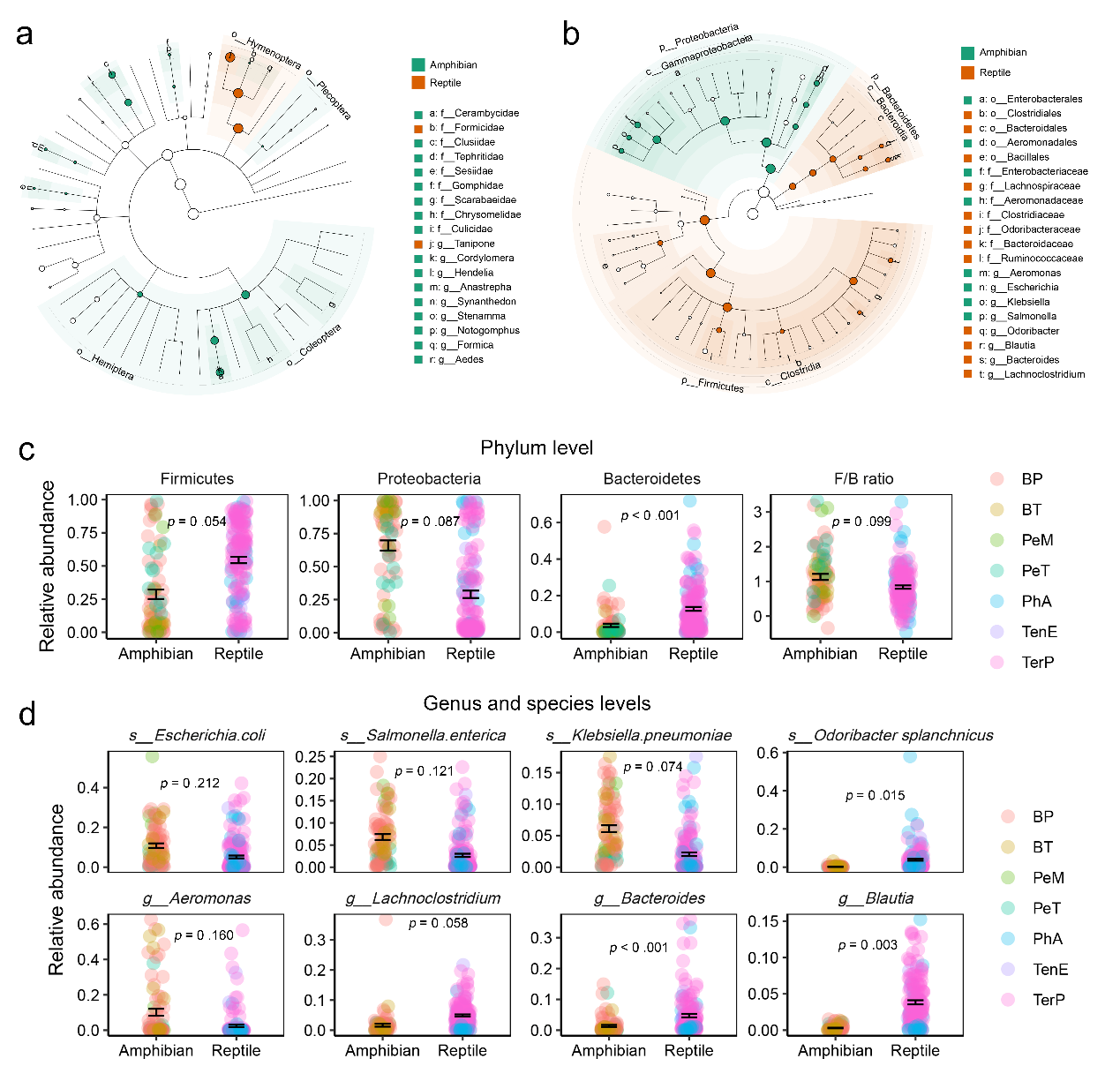


**Figure S3** Differential analyses on the gut microbiota between amphibians and reptiles. (a–b) Results of LEfSe on dietary insects (a) and gut microbiota (b) at threshold of *p* < 0.01 and LDA score > 4.0. (c–d) Dot plots presenting the relative abundance of bacterial taxa. The differences between amphibians and reptiles were examined using linear mixed models with animal class as fixed factor and species as random factor.


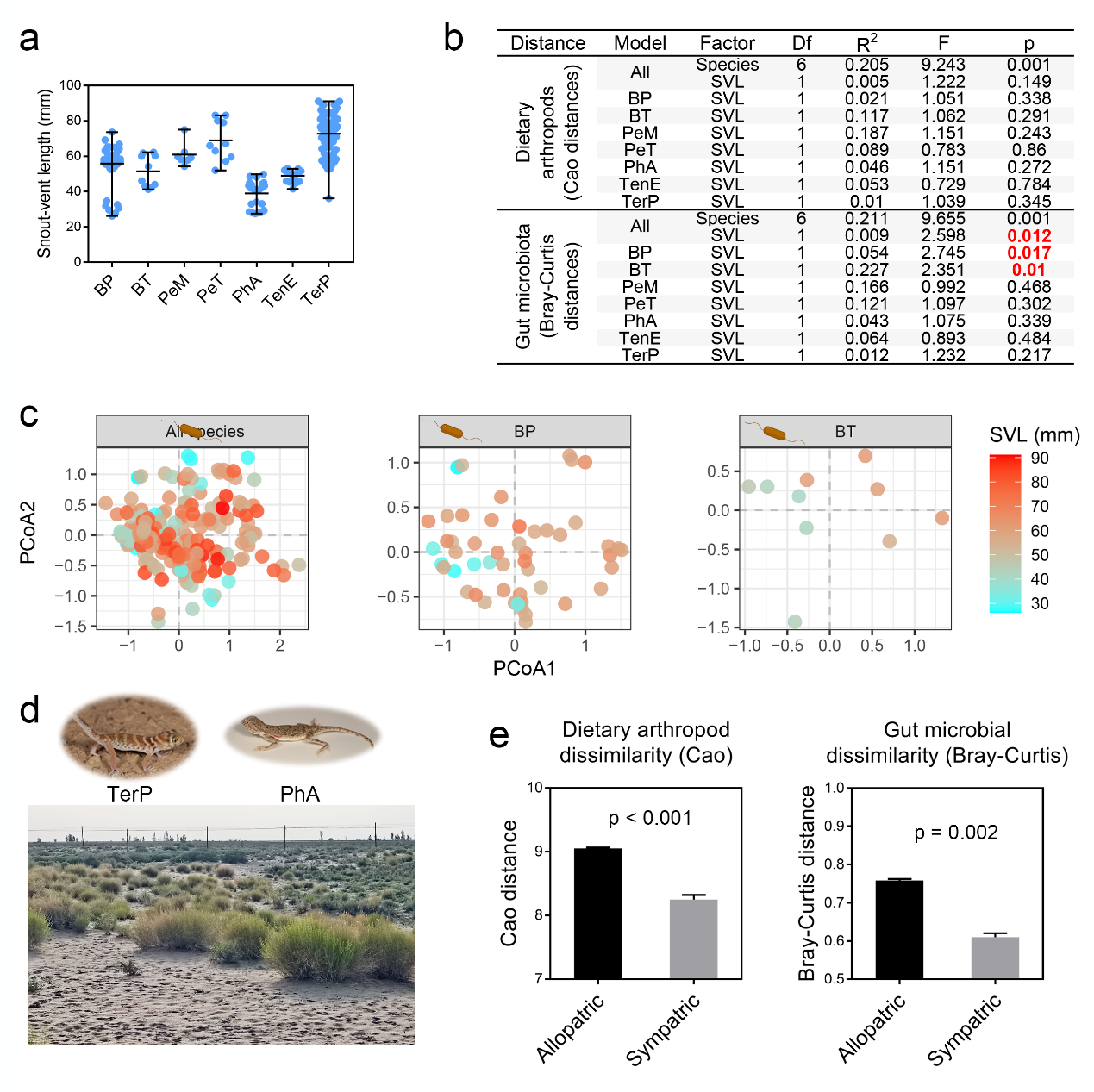


**Figure S4.** Influence of physiological and environmental factors on dietary arthropod and gut microbial composition.

(a) Snout–vent length (SVL) of each species.

(b) Effects of SVL on dietary arthropod and gut microbial composition. PERMANOVA models (across species: species and SVL as factors, using type II sums of squares with by = "margin"; within species: SVL as the factor) were applied to assess the influence of SVL on community composition. Significant effects were detected in the across-species analysis, as well as within BT and BP.

(c) PCoA scatterplots showing the SVL-dependent distribution of samples across species, and within BP and BT.

(d) Photograph of a representative site where TerP and PhA occur sympatrically (BH).

(e) Differences in dietary arthropod and gut microbial dissimilarity between sympatric and allopatric TerP–PhA pairs. Statistical comparisons were performed using Student’s *t*-test with random subsampling (500 pairs drawn each time from the total of 2704 pairs).


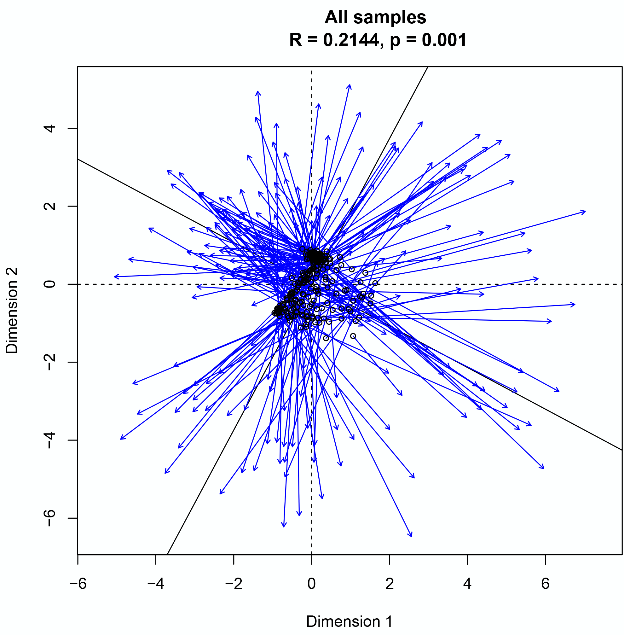


**Figure S5.** Dot plot showing the results of Procrustes analyses across all samples. Each line connects the position of a sample in the arthropod dataset (based on Cao distance) to its corresponding position in the bacterial dataset (based on Bray–Curtis distance).


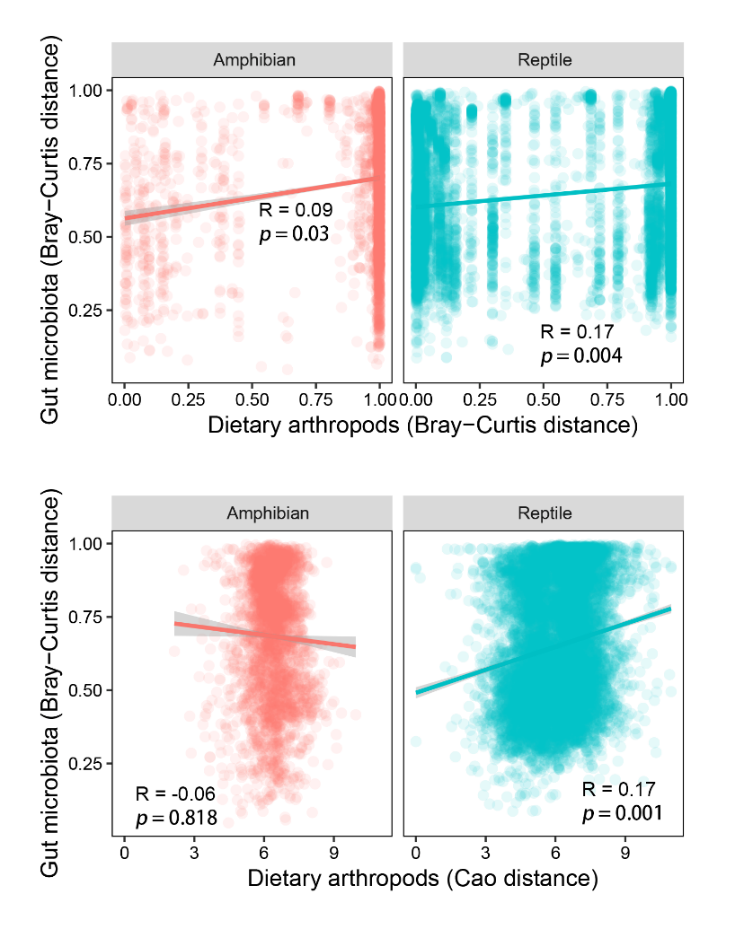


**Figure S6** Associations between gut arthropod and bacterial composition across amphibian or reptile species. *Mantel* testes (Spearman methods) were performed to examine the significance of correlations.


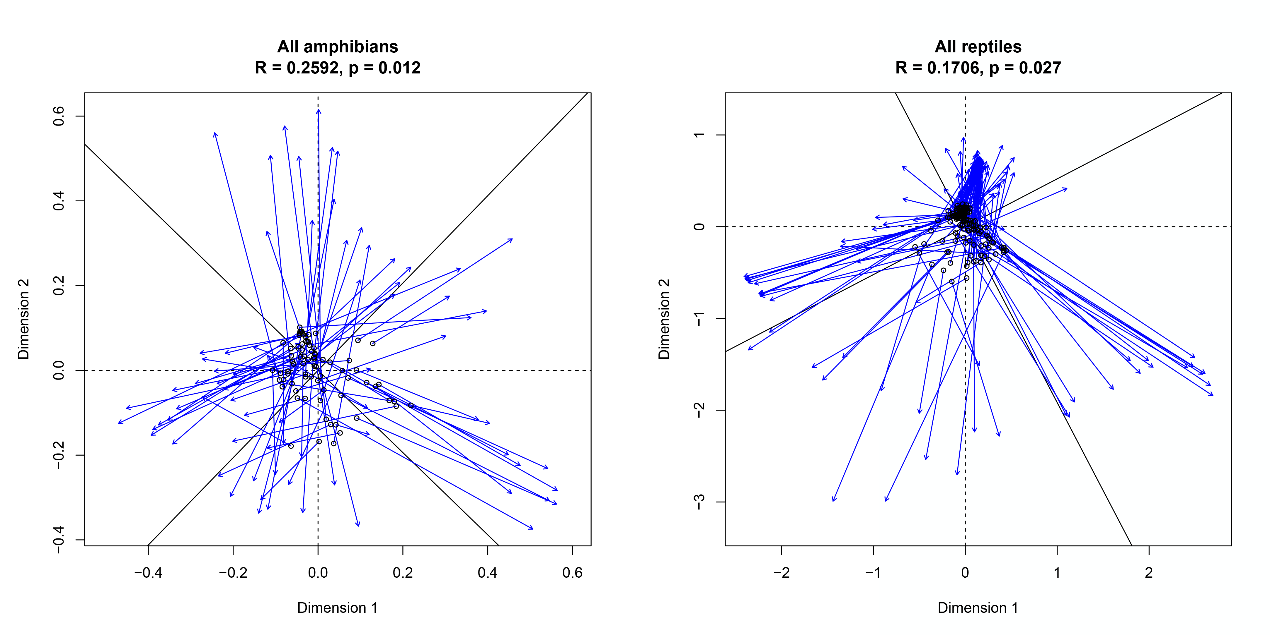


**Figure S7.** Dot plot showing the results of Procrustes analyses across all amphibian or reptile samples. Each line connects the position of a sample in the arthropod dataset (based on Cao distance) to its corresponding position in the bacterial dataset (based on Bray–Curtis distance).


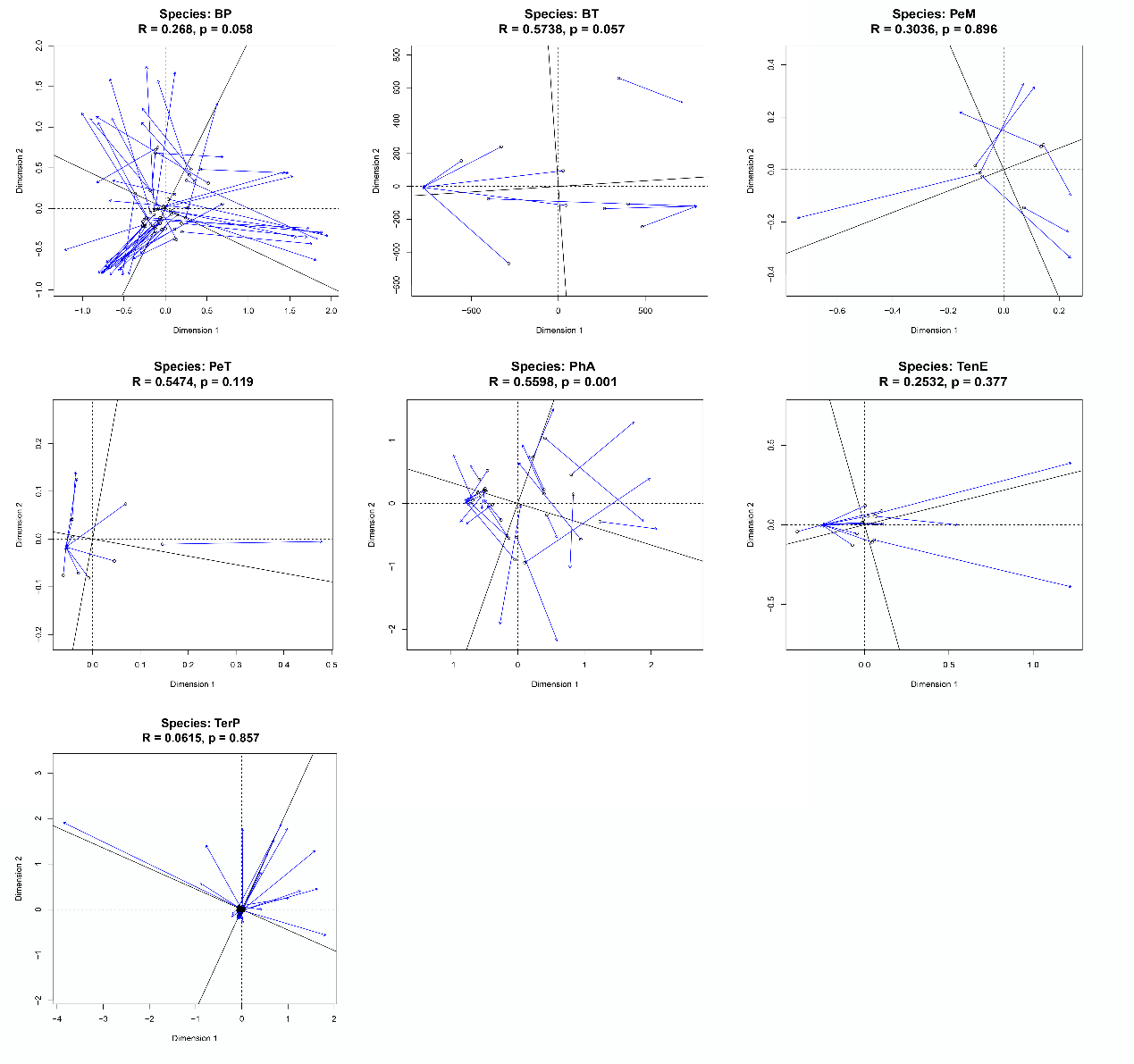


**Figure S8.** Dot plot showing the results of Procrustes analyses within each species. Each line connects the position of a sample in the arthropod dataset (based on Cao distance) to its corresponding position in the bacterial dataset (based on Bray–Curtis distance).


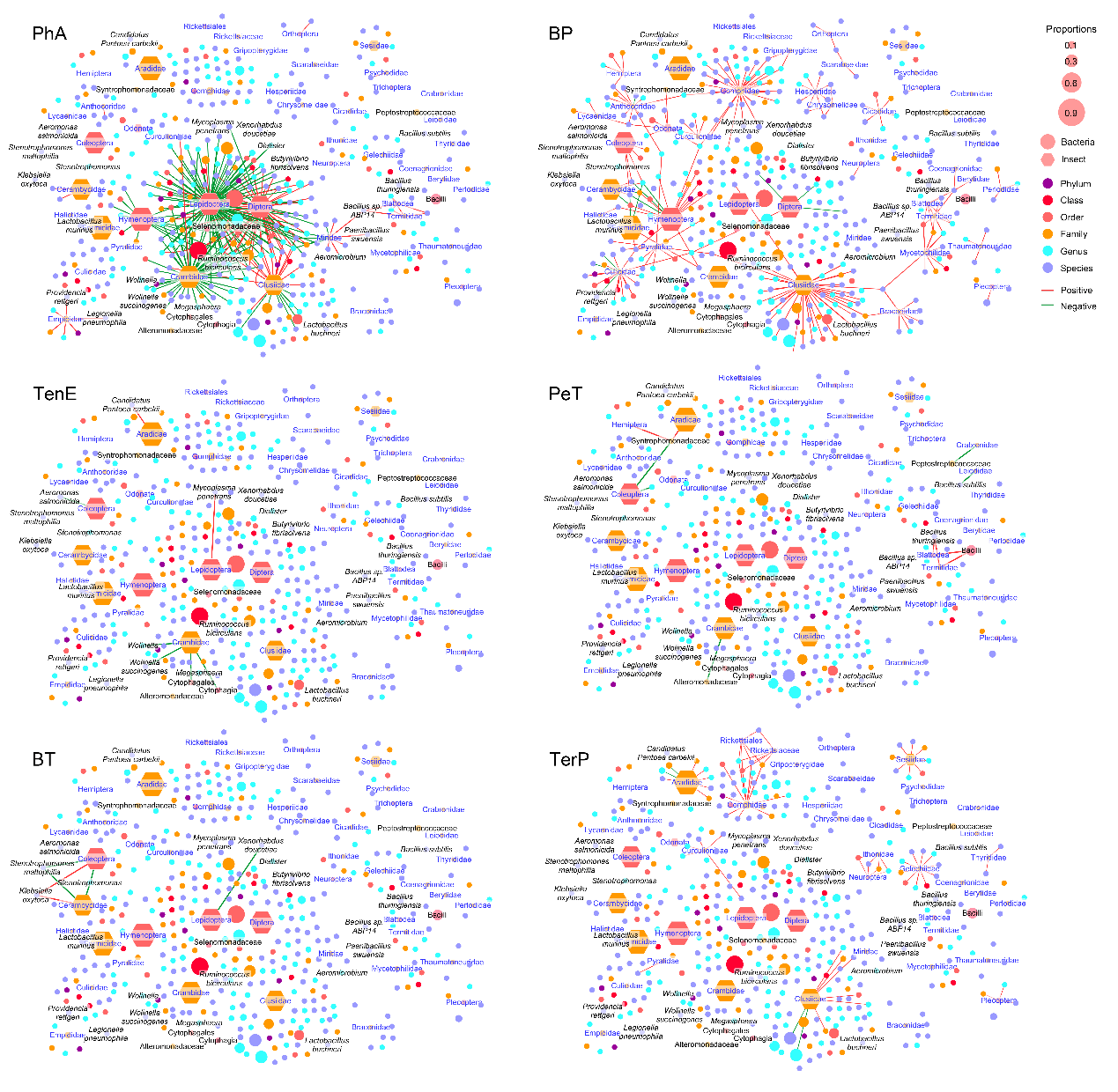


**Figure S9** Networks displaying the significant pairwise correlations between arthropod and bacterial taxa in each species. Only the correlations meeting *p* < 0.001 (Spearman correlation) and |R| > 0.4 were presented.


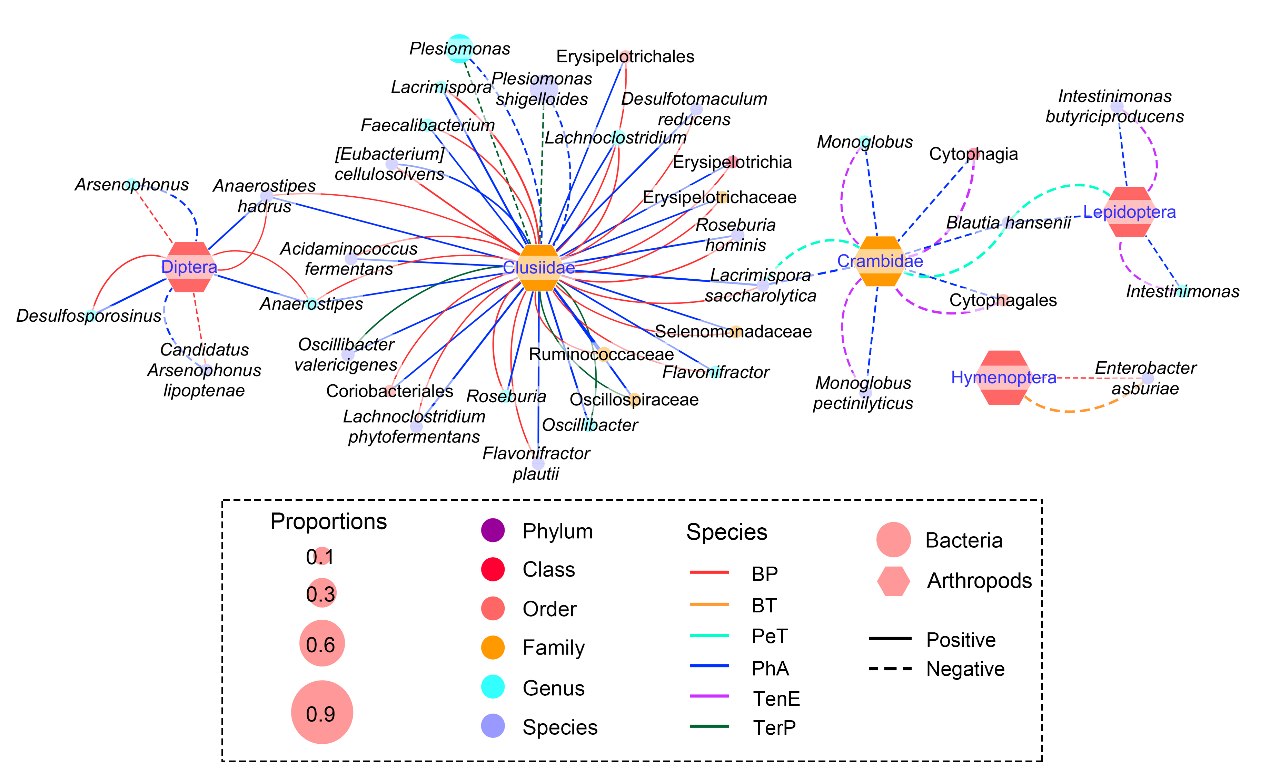


**Figure S10** (b) Network showing the significant pairwise correlations (*p* < 0.001 and |R| > 0.4) shared at least in two species.
